# EI24 suppresses TNBC tumorigenesis by regulating AKT signaling

**DOI:** 10.3389/fonc.2026.1884404

**Published:** 2026-08-06

**Authors:** Tae Wook Nam, You Min Kim, Hye Mi Park, Heeju Na, Yu-Ra Choi, Sung Gwe Ahn, Soong June Bae, Yaechan Song, Jae Hoon Lee, Joon Jeong, Han-Woong Lee

**Affiliations:** 1 Department of Biochemistry, College of Life Science and Biotechnology, Yonsei University, Seoul, Republic of Korea; 2 Gemcro, Inc., Goyang, Republic of Korea; 3 Department of Surgery, Gangnam Severance Hospital, Institute for Breast Cancer Precision Medicine, Yonsei University College of Medicine, Seoul, Republic of Korea

**Keywords:** AKT, AKT-PH domain, breast cancer, EI24, etoposide-induced gene 24, MMTV-PyMT breast cancer model, TNBC (triple-negative breast cancer)

## Abstract

**Introduction:**

Triple-negative breast cancer (TNBC) is an aggressive breast cancer subtype characterized by limited targeted therapeutic options and frequent activation of the PI3K/AKT signaling pathway. EI24, a p53-responsive tumor suppressor involved in apoptosis, autophagy, and oncogenic signaling regulation, has been implicated in multiple cancer types; however, its functional role and molecular mechanism in TNBC remain incompletely understood. This study investigated the clinical relevance of EI24 in TNBC and examined its impact on AKT signaling and tumor progression.

**Methods:**

Public breast cancer datasets and breast cancer patient samples were analyzed to assess EI24 expression patterns and clinical associations. Doxycycline-inducible EI24 overexpression systems were established in TNBC cell lines to evaluate cell proliferation and AKT pathway activity. Human phospho-kinase arrays, immunoblotting, co-immunoprecipitation, domain-mapping analyses, and immunofluorescence assays were performed to investigate the molecular mechanism underlying EI24-mediated AKT regulation. *In vivo* functional effects were examined using the MMTV-PyMT mammary tumor mouse model combined with mammary gland-specific Ei24 overexpression.

**Results:**

EI24 expression was significantly reduced in TNBC patient samples and lower EI24 expression was associated with advanced tumor progression and poorer relapse-free survival. Inducible EI24 overexpression suppressed proliferation of TNBC cell lines and reduced AKT phosphorylation at both S473 and T308. In the MMTV-PyMT model, Ei24 overexpression inhibited mammary tumor growth, reduced pulmonary metastasis, and prolonged survival. Mechanistically, EI24 interacted with the pleckstrin-homology (PH) domain of AKT and impaired growth factor-induced AKT membrane translocation, suggesting that EI24 negatively regulates AKT activation by interfering with its spatial recruitment to the plasma membrane.

**Discussion:**

These findings identify EI24 as a negative regulator of AKT signaling and support its tumor-suppressive role in TNBC progression. Our study provides mechanistic and *in vivo* evidence linking EI24 to AKT pathway regulation and supports further investigation of EI24-based approaches as potential therapeutic strategies for AKT-driven TNBC.

## 1 Introduction

Breast cancer is the most commonly diagnosed cafi 2ncer globally, with an estimated 2.3 million new cases in 2020, and its incidence is projected to exceed 3 million annually by 2040 (1–3). The treatment of breast cancer varies according to the subtypes, hormone receptors (Estrogen receptor, ER and Progesterone receptor, PR)-positive, HER2 (Human epidermal growth factor receptor 2)-positive, and TNBC (Triple-negative breast cancer), which are identified by molecular markers (4, 5). Targeted therapies for hormoe receptor-positive or HER2 positive breast cancers have greatly increased the 5-year survival rate for most breast cancer patients to nearly 90%. However, approximately 15-20% of cases, identified as TNBC, primarily depend on chemotherapy due to a lack of successful targeted therapies. This often leads to poorer outcomes compared to other breast cancer subtypes (6–8). Hence, it is crucial to delineate precise mechanisms and targets for treating individuals with TNBC.

The PI3K/AKT/mTOR signaling pathway has emerged as a critical component in the pathophysiology of TNBC (9). This pathway, which is crucial to survival, growth, and proliferation, is frequently dysregulated in TNBC (10), contributing to the malignant transformation, aggressiveness, and therapy resistance (11). The design of therapeutic agents aimed at inhibiting or modulating this pathway could potentially improve the treatment efficacy of TNBC (12). For example, alpelisib is a targeted PI3K inhibitor (13) primarily used with fulvestrant for hormone receptor-positive metastatic breast cancer (13) and is being evaluated in clinical trials for TNBC and HER2-positive breast cancer (14, 15). However, these trials are currently limited to TNBC patients with specific genetic PIK3CA or PTEN alterations (16), highlighting the need for more refined molecular subgrouping of TNBC.

EI24 (etoposide-induced gene 24) is a p53-responsive tumor suppressor gene involved in DNA damage response (17), apoptosis (18), autophagy, and regulation of oncogenic signaling pathways. EI24 suppresses tumor metastasis by binding to tumor necrosis factor receptor-associated factors 2/5 (TRAF2/5) and inhibits the non-canonical nuclear factor kappa B (NF-kB) signaling pathway (19). Furthermore, EI24 is implicated in chemoresistance in several cancer types. Knockdown of Ei24 resulted in resistance to the EGFR-tyrosine-kinase inhibitor gefitinib in non-small cell lung cancer (NSCLC) cells (20), whereas EI24 dysregulation has been implicated in esophageal squamous cell carcinoma (ESCC) growth and drug response (21, 22). EI24 is located on human chromosome 11q23-q24 (23), a genomic region frequently subject to allelic loss in multiple cancer types, including lung, ovarian, and breast cancers (24). Notably, marked downregulation of EI24 in breast cancer tissues suggests that its diminished expression could contribute to tumorigenesis and progression of breast cancer (25, 26). Recent studies have revealed that upregulated expression of microRNA-455-3p (miR-455-3p) in TNBC actively promotes cell proliferation, invasion, and migration by directly targeting EI24 (27, 28). Although these studies proposed EI24 as a possible therapeutic target and prognostic biomarker in the context of TNBC, the precise mechanism through which EI24 influences TNBC biology has not been clearly defined.

Here, we report that higher EI24 expression is associated with a favorable prognosis in TNBC patient samples. EI24 overexpression suppresses AKT phosphorylation and tumor-promoting phenotypes both *in vitro* and *in vivo*. These findings support EI24 as a candidate regulator of AKT signaling in breast cancer and provide a rationale for further investigation of EI24-restoration strategies.

## 2 Materials and methods

### 2.1 Expression comparison in normal and cancer tissue

The TNMplot web tool (www.tnmplot.com) (29) was used to compare EI24 expression between normal breast tissue and breast cancer specimens. EI24 transcript levels in primary breast cancer tissues and their association with intrinsic breast cancer subtypes were analyzed and visualized using UALCAN (http://ualcan.path.uab.edu) (30, 31).

### 2.2 Analysis of patient microarray data

Patient tumor tissue samples were surgically resected at Gangnam Severance Hospital, Yonsei University College of Medicine, Seoul, Korea, between July 1997 and December 2007. Tumor tissues (n = 298) were analyzed by microarray as previously described (32).

### 2.3 Kaplan-Meier plotter database analysis

The Kaplan-Meier Plotter database, which contains gene expression and survival data from breast cancer cohorts, was used to analyze relapse-free survival, hazard ratios, and log-rank P values. Probe 216396_s_at was used for EI24 analyses. Patients were grouped according to the auto-selected best cutoff provided by the database (33).

### 2.4 Breast cancer mouse models

All genetically engineered mice were female and 3–4 months of age. Ei24 conditional transgenic (EicTg) mice were previously described (34). EicTg mice were crossed with MMTV-PyMT and MMTV-Cre mice, which were obtained from The Jackson Laboratory (strain #002374 and #003551). All transgenic lines were maintained as heterozygotes on an FVB/N background. PyMT-induced tumors were monitored and measured weekly. Tumor volume was calculated as volume = 1/2 x length x width^2 (35, 36). Experiments were conducted according to the Korean Ministry of Food and Drug Safety (MFDS) guidelines for animal research, and protocols were approved by the Institutional Animal Care and Use Committee of Yonsei University (IACUC-A-201711-655-02). All mice were housed in a specific pathogen-free facility of the Yonsei Laboratory Animal Research Center (YLARC).

### 2.5 Genomic DNA extraction and genotyping

Mouse tail biopsies were lysed overnight at 55 °C in a buffer (50 mmol/L Tris, pH 8.0, 50 mmol/L EDTA, pH 8.0, 0.5% SDS, and proteinase K). On the following day, genomic DNA was extracted from the lysates by the phenol-chloroform method. Genotyping PCR was performed with 2X LF Taq Master Mix (Life Genomics, Korea) with the following primers: EicTg forward, 5’- ATG CAC CAG CGA TTG TCG AA -3’, reverse, 5’-GTC TTT GCT TCA TTG GCG CT-3’. MMTV-PyMT forward, 5’- GGA AGC AAG TAC TTC ACA AGG G -3’, reverse, 5’- GGA AAG TCA CTA GGA GCA GGG-3’. MMTV-Cre forward, 5’- ACC TGA AGA TGT TCG CGA TTA TCT-3’, reverse, 5’-ACC GTC AGT ACG TGA GAT ATC TT-3’. We used T-cell receptor delta chain (Tcrd) as an internal control. Internal forward, 5’-CAA ATG TTG CTT GTC TGG TG-3’ and internal reverse, 5’-GTC AGT CGA GTG CAC AGT TT-3’

### 2.6 Lung isolation and analysis of lung metastasis

Mice were euthanized by gradual CO_2_ inhalation (30–70% chamber volume displacement rate per minute) prior to tissue collection. After euthanasia, the lungs were perfused with PBS through the right ventricle with a puncture at the left ventricle, rinsed with sterile PBS, and immediately fixed in Fekete’s solution (37). Metastatic foci on the dorsal and ventral surfaces of lung lobes were divided into three categories based on foci size: under 1 mm, 1 to 2 mm, and over 2 mm. Foci were counted in triplicate, and the number was calculated as the mean.

### 2.7 Immunoblotting and antibodies

Human cells harvested in PBS and mouse tumor tissues were lysed in RIPA buffer (50 mM Tris-HCl, pH 7.5, 150 mM NaCl, 1 mM EDTA, 1% NP-40, 0.5% deoxycholic acid, 0.1% SDS, and protease inhibitors). Protein lysates were incubated with the following primary antibodies, all of which were used at a dilution of 1:1000: anti-EI24 (#42328s; Cell Signaling Technology), anti-GAPDH (sc-47724; Santa Cruz Biotechnology), anti-PDK1 (sc-515944; Santa Cruz Biotechnology), anti-phospho PDK1 (#3061s; Cell Signaling Technology), anti-PTEN (#9188; Cell Signaling Technology), anti-PP2A B subunit (#2290s; Cell Signaling Technology), anti-PP2A C subunit (#2038s; Cell Signaling Technology), anti-AKT (#9272s; Cell Signaling Technology), anti-phospho AKT (T308; #9275s; Cell Signaling Technology), anti-phospho AKT (S473; #4060S, Cell Signaling Technology), anti-GSK3αβ (sc-7291; Santa Cruz Biotechnology), anti-GSK3β (sc-11757; Santa Cruz Biotechnology), anti-mTOR (sc-8319; Santa Cruz Biotechnology), anti-phospho mTOR (sc-293132; Santa Cruz Biotechnology), anti-p70 S6K (#9202s; Cell Signaling Technology), anti-phospho p70 S6K (#9205s; Cell Signaling Technology), anti-HSP90 (sc-13119; Santa Cruz Biotechnology), anti-Actin (sc-47778; Santa Cruz Biotechnology) and anti-Tubulin (sc-166729; Santa Cruz Biotechnology). After incubation with the primary antibodies, the membranes were incubated with the appropriate peroxidase-conjugated secondary antibodies (GenDEPOT). Protein expression was detected by Amersham ImageQuant 800 Western blot imaging systems (Cytiva, Amersham, England).

### 2.8 Reagents and plasmids

Doxycycline, puromycin, and crystal violet were purchased from Sigma-Aldrich (USA). Human EI24 cDNA was subcloned into pCW57-RFP-P2A-MCS (Addgene, USA) to generate doxycycline-inducible constructs. Cells stably expressing doxycycline-inducible EI24 are referred to as inducible EI24-overexpressing (iEI24 OE) cells in this study. For transient expression and interaction analyses, EI24 cDNA was subcloned into pCS4-3xFlag for Flag-tagging, pCS4-3xHA for HA-tagging, and pEGFP-C1 to generate each tagged EI24 constructs, respectively. pLNCX-myr-HA-AKT1 was purchased from Addgene. AKT cDNA was subcloned into pCS4-3xFlag and pCS4-3xHA. AKT fragments were generated by mutagenesis-based cloning. Lipofectamine 3000 (Invitrogen, USA) was used for transfection.

### 2.9 Cell culture

Breast cancer cell lines MDA-MB-231, MDA-MB-468, and MDA-MB-453 and lung cancer cell lines H460, H358, and H1299, as well as 293T cells, were obtained from ATCC or the Korean Cell Line Bank (Seoul, Korea). Cells were maintained in DMEM or RPMI (HyClone, USA) supplemented with penicillin/streptomycin (Gibco, USA) and 10% FBS (HyClone) at 37 °C with 5% CO2 in a humidified chamber.

### 2.10 Human phospho-kinase array

MCF7 cells were transfected under normal serum-containing culture conditions with pBabe-PyMT and pCS4-3Flag-EI24. Forty-eight hours after transfection, cell lysates were analyzed using the Proteome Profiler Human Phospho-Kinase Array Kit (ARY003B; R&D Systems) according to the manufacturer’s instructions. Cell lysates were loaded onto the membranes and incubated with the antibody cocktail. After incubation with streptavidin-HRP, chemiluminescent signals were detected, and dot blots were analyzed using ImageJ.

### 2.11 Histology

Lung tissues from PyMT and PyMT-Ei24 overexpression (OE) mice were fixed in 10% formalin (#HT501640; Sigma-Aldrich) overnight. Samples were dehydrated and embedded in paraffin using an automated tissue processor (Leica TP1020; Leica Biosystems, Newcastle upon Tyne, U.K.). Sections (4-5 µm thick) were deparaffinized and stained with hematoxylin and eosin (H&E).

### 2.12 Immunofluorescence

Cells were fixed with 10% formaldehyde and permeabilized with 0.5% Triton X-100 for 10 minutes. After permeabilization, cells were incubated in blocking solution (5% bovine serum albumin in PBS with 0.5% Triton X-100) for 30 minutes at room temperature. Cells were incubated with primary antibody in blocking solution overnight at 4 °C and with Alexa Fluor 594-conjugated secondary antibody for 1 hour at room temperature. Nuclei were stained with DAPI-containing mounting medium (Abcam), and confocal images were obtained using a laser scanning confocal microscope (LSM 980; Carl Zeiss Microscopy GmbH, Jena, Germany).

### 2.13 Immunoprecipitation

Cells were harvested in PBS and lysed with Triton X-100 lysis buffer (50 mM Tris-HCl, pH7.5, 150 mM NaCl, 1mM EDTA, 0.5% Triton X-100, 0.1% sodium deoxycholate and protease inhibitors), followed by sonication. After 30 minutes of incubation on ice and centrifugation at 14000 rpm, 4 °C for 15 minutes, the whole-cell lysates were collected from the supernatant. Remaining lysates were incubated with anti-Flag magnetic beads (#A36798, Thermo Fisher Scientific) for 12 hours at 4 °C. The immunocomplexes were washed three times with lysis buffer and eluted with 2x SDS sample buffer by heating at 100 °C for 10 minutes.

### 2.14 Immunohistochemistry

Breast cancer patient tissue sections from Gangnam Severance Hospital were used for breast cancer subtyping and EI24 immunohistochemical staining. As described previously (38), 3 µm-thick tissue sections were cut from TMA blocks. After deparaffinization and rehydration using xylene and graded alcohol solutions, respectively, immunohistochemistry (IHC) was performed using a Ventana Discovery XT Automated Slide Stainer (Ventana Medical Systems, Tucson, AZ, USA).

### 2.15 Statistical analysis

The data are presented as mean ± SEM. Statistical analyses were performed using GraphPad Prism 8.0.2 software (GraphPad Software, Inc.). Two-group comparisons were performed using an unpaired Student’s t-test, and tumor growth kinetics were compared using two-way ANOVA. Cochran-Armitage trend tests were performed to evaluate linear trends in the proportion of patients with high EI24 expression across ordered T and N stage groups. Survival curves were compared using log-rank tests. Statistical significance was considered at p < 0.05.

## 3 Results

### 3.1 EI24 expression is reduced in TNBC patients and correlates with disease progression

**Figure 1:**
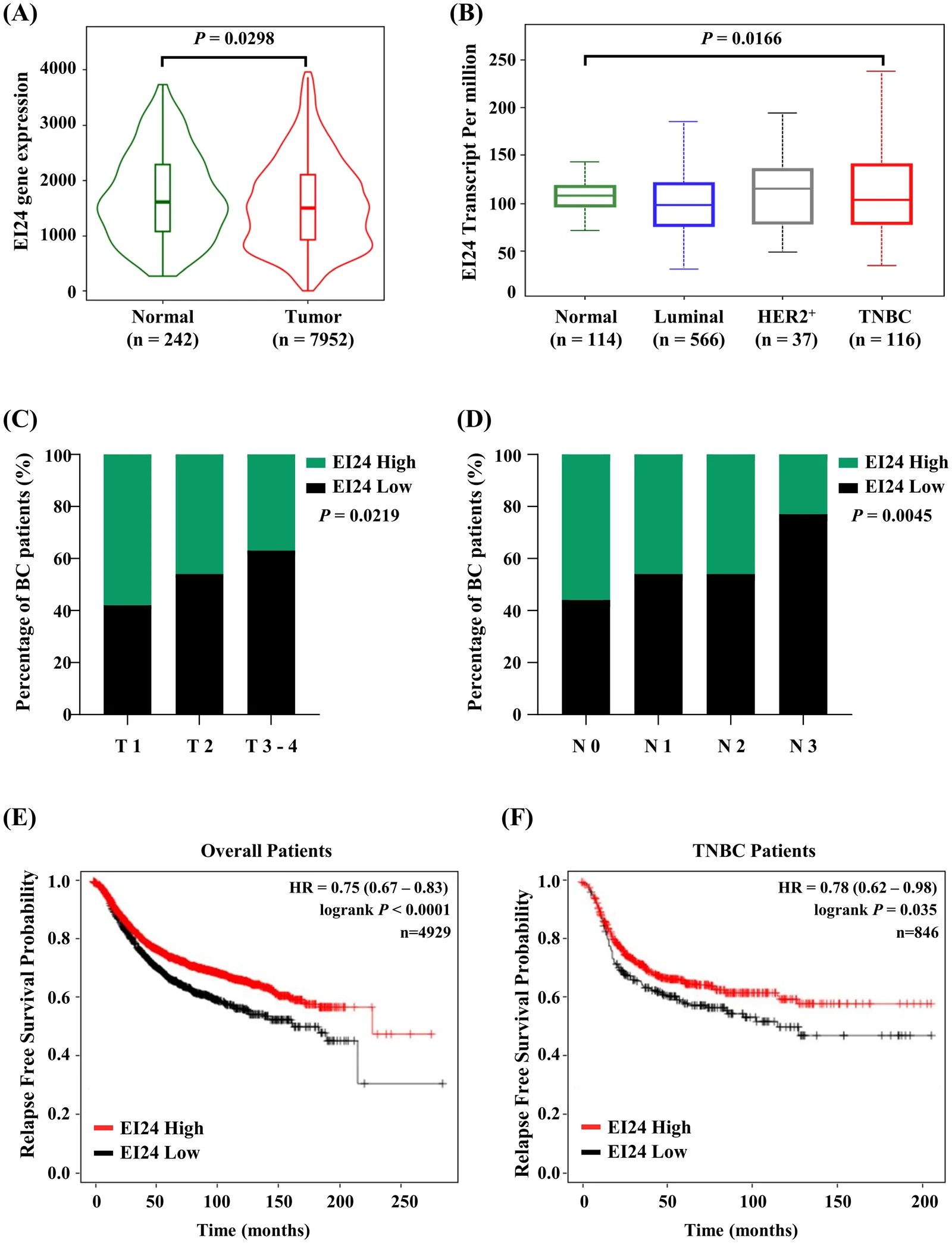
EI24 expression is reduced in breast cancer and correlates with disease progression. **(A)** TNM plot (29) demonstrated decreased EI24 expression in breast tumor tissues (n = 7952) compared with normal breast tissue (n = 242). **(B)** UALCAN analysis (30, 31) showed reduced EI24 expression in the TNBC subtype compared with other breast cancer subtypes. **(C, D)** In breast cancer patients from Gangnam Severance Hospital, the percentage of patients with low EI24 expression increased with advancing tumor stage and nodal stage. **(C)** Patient numbers by tumor stage: T1 EI24 low (n = 45), EI24 high (n = 62); T2 EI24 low (n = 90), EI24 high (n = 76); T3–4 EI24 low (n = 15), EI24 high (n = 9). **(D)** Patient numbers by nodal stage: N0 EI24 low (n = 69), EI24 high (n = 86); N1 EI24 low (n = 45), EI24 high (n = 39); N2 EI24 low (n = 19), EI24 high (n = 16); N3 EI24 low (n = 17), EI24 high (n = 5). For **Figures 1C, D**, p-values were calculated using the Cochran-Armitage trend test. **(E)** The EI24-high group showed improved relapse-free survival in all breast cancer patients (n = 4929, log-rank P < 0.0001, HR = 0.75). **(F)** In the TNBC subgroup, the EI24-high group also showed favorable relapse-free survival (n = 846, log-rank P = 0.035, HR = 0.78).

The loss of EI24 has been associated with the invasiveness of breast cancer, and its downregulation has been documented in patients with invasive ductal carcinoma (24, 25). However, the variability of EI24 expression across breast cancer subtypes and its prognostic implications have not been systematically investigated. To address this, we first assessed EI24 expression using multiple publicly available breast cancer datasets. Analysis with TNMplot (29) revealed a significant reduction in EI24 expression in breast tumors compared with normal tissues (**Figure 1A**). Furthermore, stratification by subtype using the UALCAN database (30, 31) demonstrated markedly diminished EI24 expression in TNBC relative to other subtypes (**Figure 1B**). We also examined microarray data from breast cancer patients at Gangnam Severance Hospital, classifying cases into low- and high-EI24 expression groups. As tumor and nodal stage (39) advanced, the proportion of patients with high EI24 expression declined significantly, as confirmed by Cochran-Armitage trend tests (T stage, p = 0.0219; N stage, p = 0.0045, **Figures 1C, D**). Immunohistochemical analysis of patient tissues from Gangnam Severance Hospital further supported reduced EI24 expression in TNBC compared with other breast cancer subtypes (**Table 1**). Higher EI24 expression was associated with improved relapse-free survival (RFS), particularly in TNBC and ER-positive cohorts (**Figures 1E, F**, and **Supplementary Figures 1A–C**). EI24 expression was also associated with Ki67 expression and tumor size (**Table 1**). Collectively, these findings indicate that EI24 is broadly downregulated in breast cancer, most prominently in TNBC, and support its potential value as a correlative prognostic marker in this aggressive subtype.

**Table 1:** Immunohistochemical analysis of EI24 expression levels in breast cancer patients at Gangnam Severance Hospital.

	EI24-low (n=209)	EI24-high (n=167)	*P* value
**Age**	50.10±10.20	51.43±10.08	0.209
**ER**			<0.001
Positive	75	123	
Negative	134	44	
**PR**			<0.001
Positive	64	102	
Negative	145	65	
**HER2**			n.s.
Positive	2	1	
Negative	206	166	
**Ki67**	25.43±21.34	18.26±18.31	0.001
**Tumor size**	2.40±1.49	1.95±0.82	<0.001
**Subtype**			<0.001
Luminal/HER2- ^a^	74	123	
HER2+ ^b^	2	1	
TNBC ^c^	132	43	

^a^ Luminal/HER2^-^, ER-positive and/or PR-positive and HER2-negative

^b^ HER2^+^, ER-negative, PR-negative and HER2-positive.

^c^ TNBC, ER-negative, PR-negative, and HER2-negative.

Immunohistochemical staining was performed to evaluate EI24 expression in breast cancer tissue samples from Gangnam Severance Hospital. Low EI24 expression was enriched in the TNBC subgroup and was associated with higher Ki67 levels and larger tumor size.

Continuous variables are presented as mean ± SD. Categorical variables were analyzed using Pearson’s chi-square test.

### 3.2 Elevated Ei24 expression suppresses PyMT-induced mammary tumorigenesis *in vivo*

**Figure 2:**
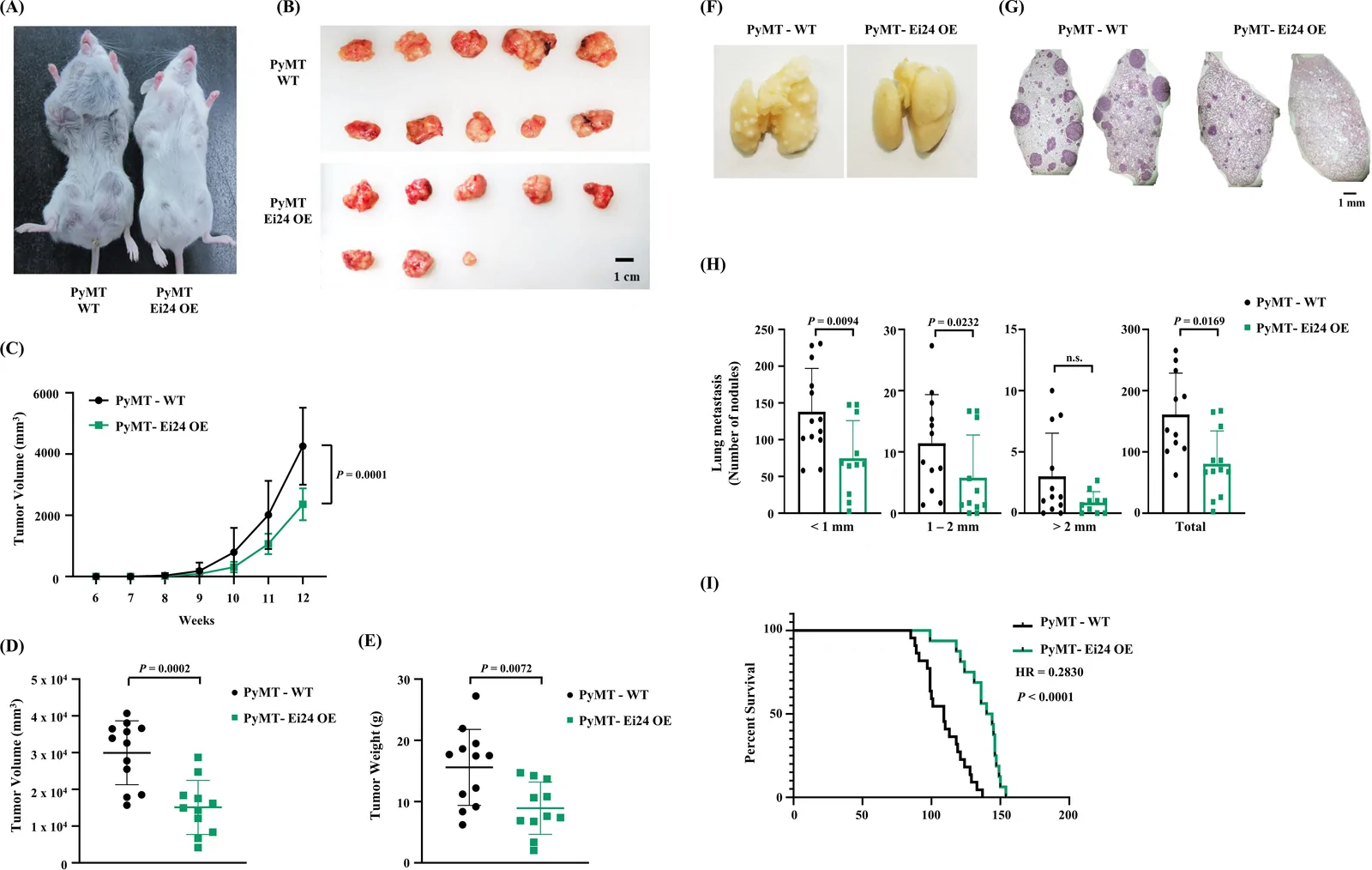
Ei24 overexpression suppresses PyMT-induced mammary tumorigenesis *in vivo*. **(A, B)** At 12 weeks of age, PyMT-Ei24 OE mice showed reduced tumor development compared with PyMT-WT mice. **(C)** Tumor growth kinetics showed suppressed tumor progression in PyMT-Ei24 OE mice (n = 6) compared with PyMT-WT mice (n = 6). Data are presented as mean ± SEM. Tumor growth kinetics in **Figure 2C** were analyzed using two-way ANOVA with the exact p-value indicated in the graph. **(D, E)** At 16 weeks of age, PyMT-Ei24 OE mice (n = 11) showed an approximately 50% reduction in tumor volume and weight compared with PyMT-WT mice (n = 12). Statistical significance was determined using an unpaired Student’s t-test, and exact p-values are indicated in the graphs. **(F–H)** The number of metastatic lung nodules in PyMT-WT (n = 12) and PyMT-Ei24 OE (n = 11) mice at 16 weeks of age. The total number was counted in whole lung tissue. Nodule size was divided into three groups (< 1 mm, 1–2 mm, and > 2 mm) based on nodule diameter, as measured with a caliper. Statistical differences between the two groups were assessed using an unpaired Student’s t-test, with exact p-values indicated in the graphs (n.s., not significant). **(I)** Overall survival was analyzed in PyMT-WT (n = 22) and PyMT-Ei24 OE (n = 16) mice. Median survival was 109 days for PyMT-WT mice and 142 days for PyMT-Ei24 OE mice, indicating a 33-day extension of median survival in PyMT-Ei24 OE mice. The log-rank test showed statistical significance (p < 0.0001, HR = 0.2830).

To define the functional role of EI24 in breast cancer development *in vivo*, we used the MMTV-PyMT transgenic mouse model, in which the expression of polyomavirus middle T antigen (PyMT) is driven by the mouse mammary tumor virus (MMTV) promoter, resulting in highly aggressive and metastatic mammary tumors (40). To assess the impact of Ei24 overexpression in this model, we generated triple transgenic mice by crossing Ei24 conditional transgenic (EicTg) mice (34) with MMTV-Cre (41) and MMTV-PyMT mice, yielding MMTV-PyMT; MMTV-Cre; EicTg (PyMT-Ei24 OE) offspring (**Supplementary Figure 2A**). These mice showed elevated Ei24 expression in mammary tumors, whereas PyMT expression was comparable between PyMT-WT and PyMT-Ei24 OE mice (**Supplementary Figure 2B**). At 12 weeks of age, PyMT-Ei24 OE mice displayed reduced tumor incidence and burden compared with PyMT-WT controls (**Figures 2A–C**). By 16 weeks, tumors in PyMT-Ei24 OE mice showed an approximately 50% reduction in both volume and weight relative to control mice (**Figures 2D, E**). Given that lung metastasis is frequently observed in MMTV-PyMT mice (40), we further analyzed pulmonary lesions at 16 weeks and observed a marked decrease in the number of metastatic nodules on the lung surface in the PyMT-Ei24 OE group (**Figures 2F–H**). Consistent with these antitumor effects, overall survival was significantly extended in PyMT-Ei24 OE mice (**Figure 2I**). Collectively, these findings indicate that elevated Ei24 expression suppresses tumorigenesis and metastatic dissemination in the MMTV-PyMT model, supporting EI24 as a functional suppressor of aggressive mammary tumor progression.

### 3.3 EI24 overexpression reduces the proliferation of TNBC cell lines

**Figure 3:**
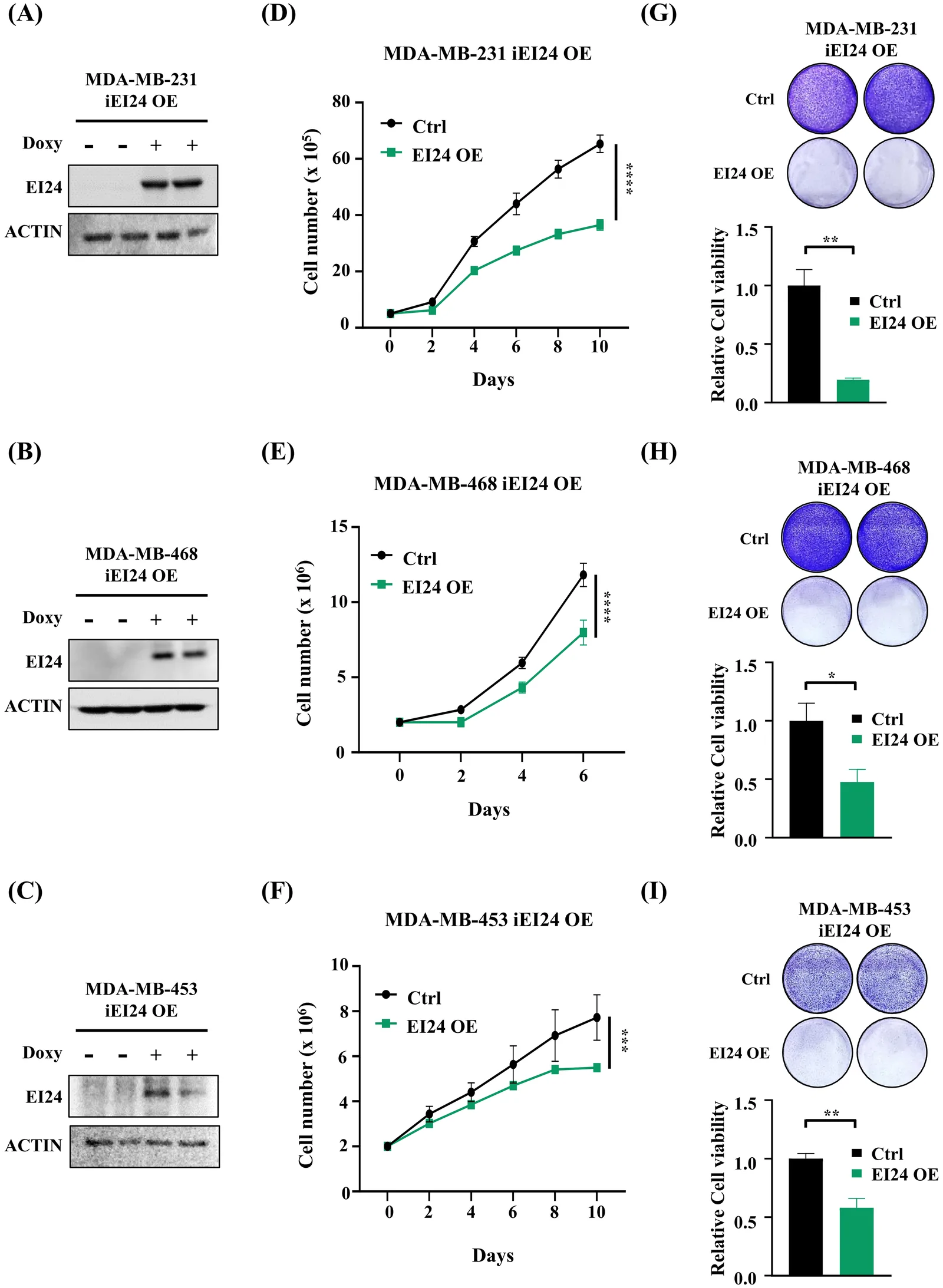
EI24 overexpression inhibits proliferation of TNBC cell lines. **(A–C)** Doxycycline-inducible GFP-tagged EI24 overexpression cell lines were established using MDA-MB-231, MDA-MB-468, and MDA-MB-453 cells. GFP-tagged EI24 expression was confirmed by Western blotting using an anti-EI24 antibody after doxycycline treatment (1 µg/ml, 24 hours). ACTIN was used as a loading control. **(D–F)** EI24-overexpressing cell lines showed suppressed cell growth rates. **(D)** MDA-MB-231 cells were seeded at 0.5 million cells and incubated for ten days with doxycycline treatment. Cells were counted every two days. **(E)** MDA-MB-468 cells were seeded at two million cells and incubated for six days with doxycycline treatment. Cells were counted every two days. **(F)** MDA-MB-453 cells were seeded at two million cells and incubated for ten days with doxycycline treatment. Cells were counted every two days (*** p < 0.001, **** p < 0.0001). **(G–I)** Crystal violet staining further showed that EI24 overexpression inhibited cell growth compared with control groups. Cells were incubated for three days with doxycycline treatment (*p < 0.05, **p < 0.01).

To investigate the influence of EI24 on TNBC cell growth, we generated doxycycline-inducible EI24 overexpression (iEI24 OE) cell lines using MDA-MB-231, MDA-MB-468, and MDA-MB-453 cells (**Figures 3A–C**). Upon induction, all iEI24 OE cell lines exhibited reduced growth rates compared with their respective control groups (**Figures 3D–F**). These observations align with previous reports indicating that upregulation of microRNA-455-3p, a negative regulator of EI24, leads to enhanced TNBC cell proliferation (27). In addition, crystal violet staining assays confirmed that EI24 overexpression substantially impeded cell growth relative to control groups (**Figures 3G–I**). These results support the notion that EI24 expression is functionally linked to cellular proliferation in TNBC models.

### 3.4 EI24 inhibits AKT phosphorylation in TNBC

**Figure 4:**
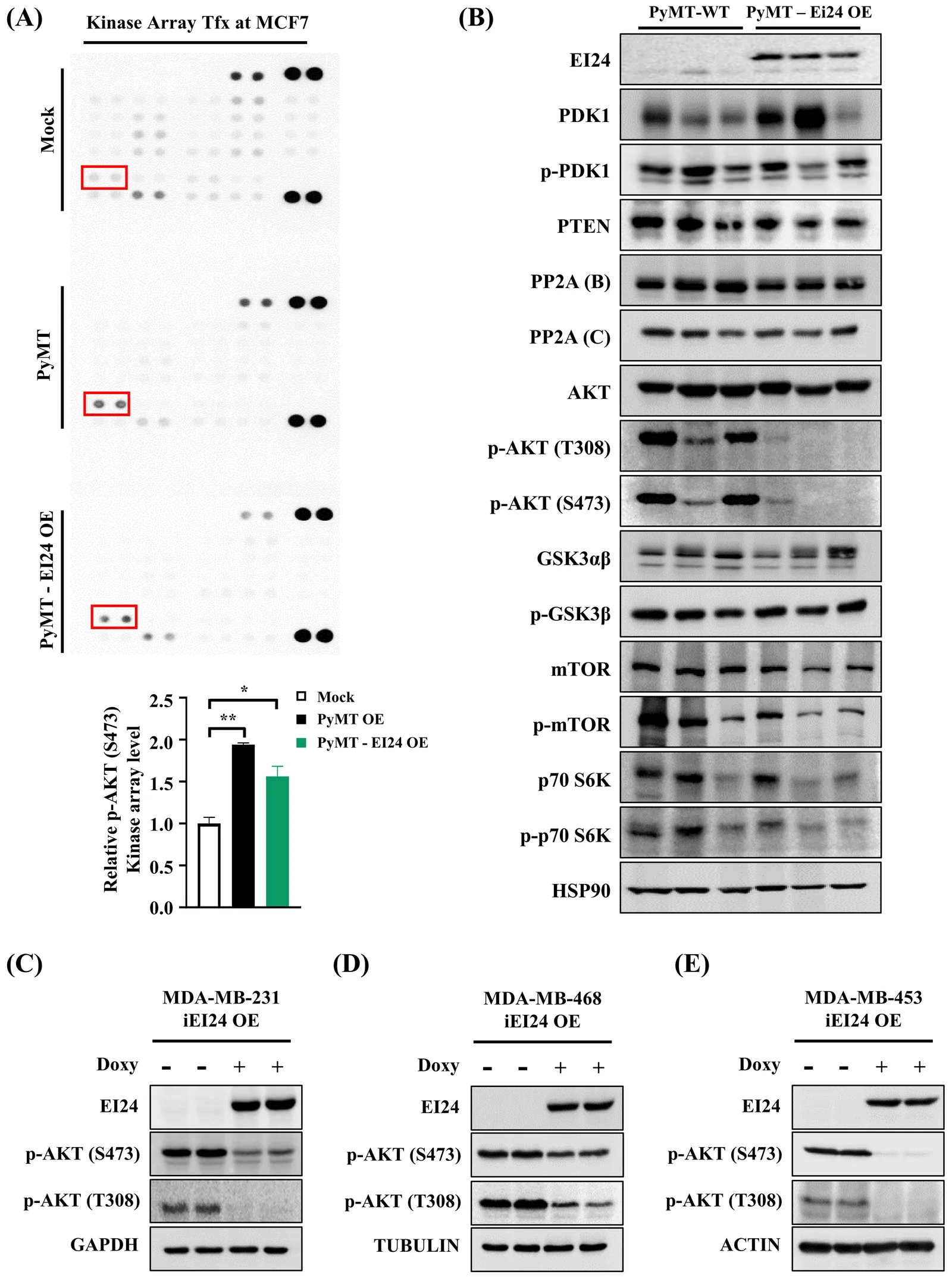
EI24 suppresses AKT phosphorylation in TNBC. **(A)** MCF7 cells were transfected with PyMT and EI24 cDNA. A Human Phospho-Kinase Array was performed using MCF7 cell lysates after transfection. The red box indicates the dot blots corresponding to AKT phosphorylation at serine 473 (p-AKT S473). Relative p-AKT (S473) levels were increased by PyMT overexpression and reduced by EI24 overexpression. **(B)** Western blot analysis of PI3K/AKT signaling in breast tumors from 12-week-old PyMT-WT (n = 3) and PyMT-Ei24 OE (n = 3) mice. Ei24 overexpression in breast tumors resulted in reduced levels of p-AKT T308 and p-AKT S473. Phosphorylated mTOR and p70 S6K levels were also decreased, whereas GSK3β phosphorylation was not clearly changed. Upstream AKT regulators, including PDK1, PTEN, and PP2A family members, were not detectably altered. HSP90 was used as a loading control. **(C–E)** Western blot analysis of p-AKT S473 and T308 in doxycycline-inducible EI24 overexpression cell lines. Doxycycline (1 µg/ml) was administered for 48 hours. Representative blots from duplicate samples are shown. **(C)** MDA-MB-231 iEI24 OE cells; GAPDH was used as a loading control. **(D)** MDA-MB-468 iEI24 OE cells; TUBULIN was used as a loading control. **(E)** MDA-MB-453 iEI24 OE cells; TUBULIN was used as a loading control. *P < 0.05; **P < 0.01.

To explore the molecular mechanism underlying EI24-mediated suppression of PyMT-induced tumorigenesis and diminished cell growth in TNBC cell lines, we performed a proteome profiler human phospho-kinase array. Lysates from MCF7 cells transfected with PyMT alone or co-transfected with PyMT and EI24 (PyMT-EI24 OE) were analyzed. Overexpression of PyMT (**Supplementary Figure 3A**) increased phosphorylation of multiple kinases (**Figure 4A**), most notably AKT at serine 473 (p-AKT S473), a key downstream event in PyMT-driven PI3K/AKT signaling (42, 43). In contrast, PyMT-EI24 OE cells displayed decreased levels of p-AKT S473, which was further confirmed by Western blot analyses (**Figure 4A** and **Supplementary Figure 3B**). We further examined whether EI24 similarly regulates AKT phosphorylation *in vivo* using breast tumors from PyMT mice. Consistent with our *in vitro* data, p-AKT levels were significantly elevated in PyMT tumors relative to normal mammary tissues from WT mice (**Supplementary Figure 3C**). Notably, EI24 overexpression in these tumors reduced phosphorylation of AKT at both S473 and threonine 308 (T308) (**Figure 4B**), indicating attenuation of AKT activation at multiple regulatory sites. Similar results were observed in TNBC cell lines in which EI24 overexpression reduced proliferation; these cells also exhibited reduced AKT phosphorylation (**Figures 4C–E**). To determine whether this mechanism is conserved across cancer types, we overexpressed EI24 in lung cancer cell lines (H1299, H358, and H460). Unlike TNBC cells, EI24 did not consistently alter AKT phosphorylation in these models (**Supplementary Figure 3D**), suggesting that the regulatory effects of EI24 on AKT activity may be cell-type or context dependent. Collectively, these findings indicate that EI24 suppresses TNBC cell proliferation at least in part by attenuating AKT phosphorylation, thereby modulating a key pathway involved in tumor cell growth and survival.

### 3.5 EI24 impairs AKT membrane translocation through interaction with the AKT PH domain

To investigate the mechanisms underlying the observed alterations in AKT phosphorylation, we examined the upstream regulators of AKT activation. Unexpectedly, EI24 overexpression did not alter the levels of key kinases or phosphatases that directly modulate p-AKT, including PDK1 or PTEN (**Figure 4B**), suggesting that EI24 regulates AKT phosphorylation through an alternative mechanism independent of these canonical upstream effectors.

**Figure 5:**
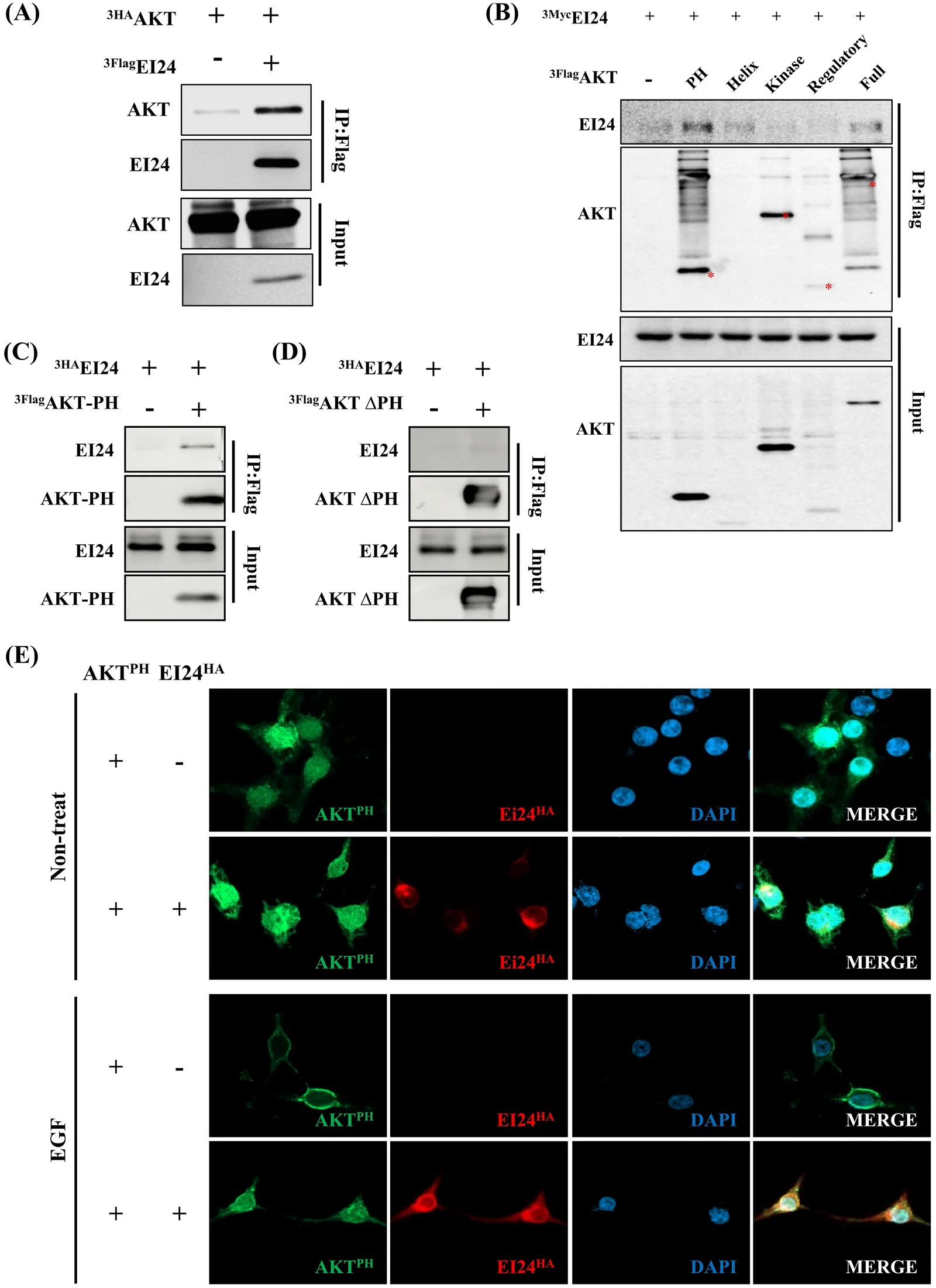
EI24 inhibits AKT translocation through interaction with the PH domain and suppresses AKT activation. **(A–D)** Immunoprecipitation (IP)-Western blot analysis was performed to examine the interaction between EI24 and AKT. **(A)** Lysates were pulled down using Flag-tagged EI24 and blotted for AKT. **(B)** Domain mapping of AKT was performed using EI24. Flag-tagged AKT fragments and full-length AKT were pulled down. **(C, D)** Fragment IP analyses supported the interaction of EI24 with the PH domain of AKT. **(E)** Immunofluorescence analysis was used to examine AKT-PH-GFP translocation in HEK293 cells after transfection with AKT-PH-GFP and EI24. Alexa Fluor 594 was used to detect 3HA-tagged EI24. Cells were treated with EGF (100 ng/ml) for 15 minutes after overnight serum starvation.

**Figure 6:**
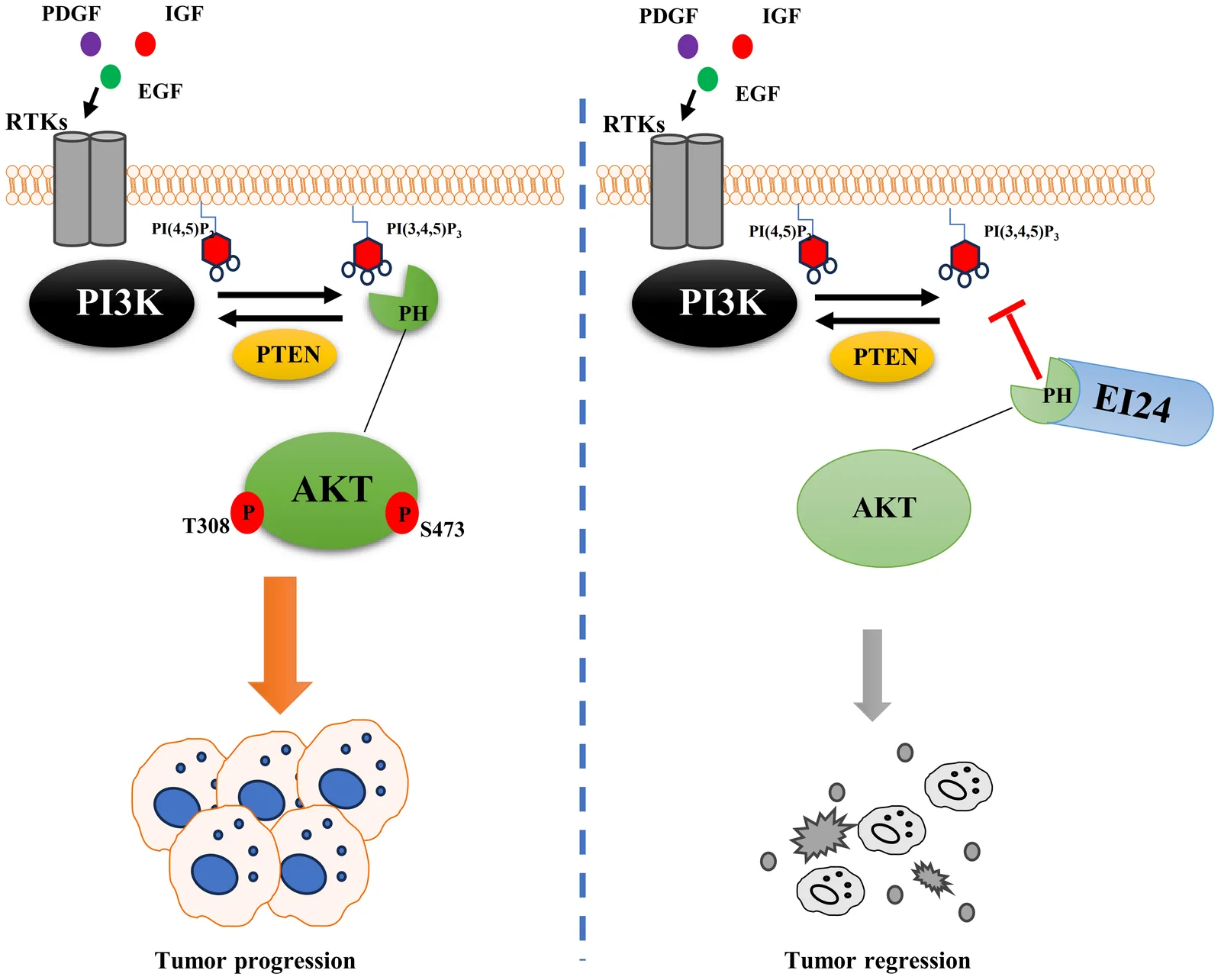
Schematic representation of tumor suppression by EI24. EI24 overexpression is proposed to hinder AKT translocation to the plasma membrane, thereby attenuating AKT pathway activation and TNBC progression.

Subsequently, immunoprecipitation (IP) assays demonstrated a robust interaction between EI24 and AKT, as confirmed by co-IP Western blot analysis (**Figure 5A**). To map the domain responsible for this interaction, we tested four AKT fragments and identified the pleckstrin-homology (PH) domain as the primary region associated with EI24 binding (**Figure 5B**). This interaction was further supported by co-IP experiments using the isolated PH domain fragment and an AKT mutant lacking the PH domain (**Figures 5C, D**). The PH domain of AKT is crucial for its activation because it mediates binding to phosphatidylinositol (3–5, 4, 5)-trisphosphate (PIP3) at the plasma membrane. Engagement of PIP3 with the PH domain induces a conformational change that allows AKT to translocate to the membrane, where it undergoes phosphorylation at T308 and S473 (44). Hence, we hypothesized that EI24-mediated regulation of p-AKT occurs predominantly through its interaction with the PH domain, which impairs AKT translocation to the plasma membrane. To test this hypothesis, we performed immunocytochemistry (ICC) to monitor the localization of the AKT-PH domain following PI3K/AKT pathway stimulation by epidermal growth factor (EGF) (45). Under basal conditions, the AKT-PH domain was diffusely distributed in the cytoplasm, while EGF treatment induced its translocation to the plasma membrane (**Figure 5E**). However, EI24 overexpression reduced this EGF-induced membrane translocation of AKT-PH (**Figure 5E**). Taken together, these findings support a model in which EI24 interacts with the PH domain of AKT, thereby limiting AKT membrane recruitment and subsequent phosphorylation (**Figure 6**). This mechanism provides a molecular explanation for EI24-mediated suppression of AKT signaling in breast cancer.

## 4 Discussion

The treatment of TNBC remains challenging due to the lack of actionable molecular targets and the heavy reliance on chemotherapy (8, 9). Consequently, there is a critical need for treatment strategies capable of effectively halting the undesired processes driving TNBC. Given that the PI3K/AKT pathway is frequently activated in TNBC and contributes to its aggressive phenotype, identifying regulators of AKT may provide new therapeutic opportunities (11). In this study, we show that EI24 functions as a suppressor of TNBC progression by attenuating AKT activation.

Clinically, EI24 expression was reduced in TNBC patients, and lower EI24 levels were associated with advanced tumor stage and poorer relapse-free survival (**Figure 1**). Functionally, increasing EI24 expression inhibited proliferation of multiple TNBC cell lines and significantly suppressed tumor growth and metastasis in the MMTV-PyMT model (**Figures 2**, **3**). Although the MMTV-PyMT model does not fully represent all molecular subtypes of TNBC, late-stage MMTV-PyMT tumors and derived Met-1 cells have been reported to display TNBC-relevant features, including ER/PR-negative and AR-positive characteristics (46). Thus, the TNBC cell-line data provide direct mechanistic evidence in TNBC models, whereas the MMTV-PyMT data support the tumor-suppressive function of EI24 in aggressive mammary tumor progression with TNBC-relevant features. The reduction of EI24 in TNBC may be partly explained by miR-455-3p-mediated post-transcriptional repression, as previous studies have shown that miR-455-3p is upregulated in TNBC and directly targets EI24 (27, 28). However, whether EI24 protein stability is regulated by proteasome-dependent degradation or other post-translational mechanisms in TNBC remains to be determined.

Mechanistically, our data support a model in which EI24 associates with the PH domain of AKT and impairs its membrane translocation, a prerequisite for phosphorylation at T308 and S473. Unlike classical PI3K and AKT inhibitors, EI24 did not alter upstream regulators such as PTEN or PDK1, suggesting that EI24 may exert its effect at the spatial activation step rather than through canonical upstream modulation. Notably, not all downstream targets responded uniformly: GSK3β phosphorylation remained unchanged despite reduced AKT phosphorylation, reflecting potential substrate-specific regulation. This discrepancy may arise from the distinct subcellular localization of AKT substrates or the involvement of compensatory kinase pathways that maintain GSK3 β phosphorylation.

Although EI24 effectively suppressed AKT signaling in TNBC and PyMT tumors, its effects were inconsistent across lung cancer cell lines, suggesting that the EI24-AKT axis may operate in a tumor type-specific or AKT-dependency-specific manner. This variability may reflect differences in basal AKT activity, intrinsic AKT dependency, or the presence of robust compensatory signaling pathways across cancer types. Consequently, EI24-mediated inhibition of AKT membrane recruitment may be more pronounced in highly AKT-dependent tumors, whereas cells with lower AKT reliance or active feedback networks may remain less responsive to EI24 overexpression. These findings support a tumor-suppressive role of EI24 in TNBC, while also suggesting that EI24 function may vary depending on cancer type and cellular context. This context-dependent role is further supported by a recent review describing EI24 as a tumor suppressor in several malignancies, including breast cancer, while also highlighting its pro-tumorigenic properties in selected tumor contexts (47). Consistent with this broader view, a pan-cancer analysis further reported that EI24 expression is associated with patient prognosis and immune infiltration characteristics across multiple cancer types, supporting the broader clinical relevance of EI24 beyond TNBC (48). In breast cancer, including our TNBC models, EI24 appears to behave as a tumor suppressor that constrains proliferation and metastatic progression. In contrast, in pancreatic ductal adenocarcinoma, EI24 has been reported to exert both tumor-suppressive effects through c-Myc regulation and pro-tumorigenic functions linked to its role in autophagy, indicating that EI24 can either restrain or facilitate tumor growth depending on the cellular context (49, 50). Furthermore, experimental skin carcinogenesis models have shown that loss of EI24 attenuates PKCα signaling and reduces DMBA-induced tumor formation, suggesting a tumor-promoting function of EI24 in the skin (51). Thus, EI24 can function as either a tumor suppressor or a tumor promoter depending on tissue type, oncogenic drivers, and signaling context. In TNBC, our data position EI24 as a tumor-suppressive regulator of AKT signaling. Strategies aimed at increasing EI24 expression, such as modulating miRNAs that repress EI24 or enhancing EI24 protein stability, may complement existing PI3K/AKT inhibitors and potentially improve their therapeutic efficacy.

Subtype-specific interpretation of our findings requires careful consideration. Although this study focuses on TNBC, the subtype-stratified survival analyses indicate that the prognostic relevance of EI24 may not be entirely restricted to TNBC. Higher EI24 expression was associated with favorable RFS in the Luminal A cohort as well as in TNBC, whereas this association was not significant in Luminal B or HER2-positive cohorts. In addition, the TNBC cell lines used in this study represent distinct molecular contexts within the TNBC spectrum, including mesenchymal/mesenchymal stem-like features in MDA-MB-231, basal-like model features in MDA-MB-468, and MDA-MB-453 as a luminal androgen receptor/molecular apocrine-like breast cancer cell line with androgen receptor-associated features (52–54).

This study also has several limitations. First, the current data rely primarily on gain-of-function models and do not include complementary loss-of-function or rescue experiments. The low endogenous EI24 expression in the TNBC models used in this study may limit the dynamic range and interpretability of further knockdown or knockout experiments. Future loss-of-function studies using EI24-high breast cancer models, non-TNBC breast cancer subtypes, or patient-derived organoid systems will be instrumental to further clarify the physiological role of EI24 function in breast cancer. Second, co-IP and domain-mapping experiments support an association between EI24 and the AKT PH domain; however, purified biochemical binding assays or structural analyses will be required to establish direct binding and define the EI24-PH interface. Third, the clinical analyses are correlative and do not include multivariable Cox regression adjusting for potential confounding factors, including tumor stage, nodal status, ER/PR/HER2 status, Ki67, and molecular subtype. Although Kaplan-Meier analyses demonstrate a strong prognostic trend, multivariable models will be required in larger cohorts to formally establish EI24 as an independent prognostic marker. Finally, while the PyMT model recapitulates key aspects of aggressive and metastatic mammary tumor progression, it does not fully capture the heterogeneity of human TNBC. Future studies will be needed to clarify how EI24 integrates into broader signaling networks and to validate its therapeutic potential in genetically defined TNBC subsets. Overall, our findings support a model in which EI24 suppresses AKT signaling and tumor-promoting phenotypes by limiting AKT membrane recruitment and activation, highlighting the potential of EI24-based strategies for targeting AKT-driven breast cancers.

## Data Availability

The datasets presented in this study can be found in online repositories. The names of the repository/repositories and accession number(s) can be found in the article/**Supplementary Material**.
