# Supplementary material for: EI24 suppresses TNBC tumorigenesis by regulating AKT signaling
Source: Front Oncol. 2026 Aug 6;16:1884404. doi: 10.3389/fonc.2026.1884404 (PMC13489907; doi:10.3389/fonc.2026.1884404)
Supplement: Supplementary file 1 [file SupplementaryFile1.docx]

**Supplementary Figure Legends**

**Supplementary Figure 1. Relapse-free survival according to EI24 expression in other breast cancer subtypes.**

(A) In the Luminal A subtype, which is generally ER-positive, the EI24-high group showed improved relapse-free survival (n = 2277, log-rank P < 0.0001, HR = 0.64). (B, C) In contrast, Luminal B and HER2-positive subtypes did not show a statistically significant difference in relapse-free survival between EI24-high and EI24-low groups. (B) Luminal B subtype (n = 1491, log-rank P = 0.16, HR = 0.87). (C) HER2-positive subtype (n = 315, log-rank P = 0.09, HR = 1.39).

**Supplementary Figure 2. Validation of Ei24 overexpression in MMTV-PyMT mammary gland-specific transgenic mice.**

(A) Genotyping PCR products for EicTg, MMTV-PyMT, MMTV-Cre, and the T-cell receptor delta chain (Tcrd) internal control are shown. The expected amplicon sizes are indicated in the figure. (B) Ei24 mRNA expression was increased in PyMT-Ei24 OE breast tumors, whereas PyMT expression was not significantly different between PyMT-WT and PyMT-Ei24 OE tumors (both groups, n = 3). (** p < 0.01)

**Supplementary Figure 3. EI24 suppresses PyMT-induced AKT phosphorylation.**

(A, B) MCF7 cells were transfected with PyMT and EI24 cDNA. (A) PyMT and EI24 overexpression levels were confirmed by qPCR in transfected MCF7 cells. (B) Western blot analysis confirmed the kinase array result, showing that EI24 reduced PyMT-induced AKT phosphorylation. (C) Protein levels of AKT and phosphorylated AKT were compared between mammary glands of FVB-WT mice (n = 4) and breast tumors of 12-week-old MMTV-PyMT mice (n = 4). Consistent with previous studies (45, 46), AKT phosphorylation at serine 473 (S473) and threonine 308 (T308) was increased in PyMT tumors compared with WT mammary glands. Actin was used as a loading control. (D) Western blot analysis of AKT and p-AKT (S473) in lung cancer cell lines H1299, H358, and H460. Cells were harvested 48 hours after transient transfection of EI24. Actin was used as a loading control.
